# Managing gastrointestinal challenges: Diarrhea in sickle cell anemia

**DOI:** 10.1097/MD.0000000000038075

**Published:** 2024-05-03

**Authors:** Emmanuel Ifeanyi Obeagu, Getrude Uzoma Obeagu

**Affiliations:** a Department of Medical Laboratory Science, Kampala International University, Uganda; b School of Nursing Science, Kampala International University, Uganda.

**Keywords:** diarrhea, gastrointestinal challenges, management, sickle cell anemia

## Abstract

Sickle cell anemia (SCA), a hereditary hemoglobinopathy, is characterized by the presence of abnormal hemoglobin and has long been associated with a wide range of complications. While much attention has been given to the condition hematological aspects, gastrointestinal complications, particularly diarrhea, have been relatively understudied and often overlooked. This publication delves into the management of gastrointestinal challenges, with a focus on diarrhea, in individuals living with SCA. The pathophysiology of SCA is intrinsically linked to gastrointestinal complications, and diarrhea is a common manifestation of this condition. This abstract publication outlines the key elements discussed in the full-length work, which includes the clinical presentation of diarrhea in these patients, the diagnostic tools used to evaluate the condition, and various management strategies to alleviate symptoms and enhance the overall quality of life for affected individuals. The paper emphasizes the importance of patient education, offering healthcare professionals valuable insights into how to inform and support patients in managing their conditions effectively. It also highlights the need for continued research to further our understanding of gastrointestinal challenges in SCA and to identify potential areas for future therapeutic interventions. Ultimately, the comprehensive management of diarrhea in individuals with SCA is vital for their overall well-being. This publication serves as a valuable resource for healthcare providers, researchers, and caregivers in addressing the gastrointestinal challenges that accompany SCA, ultimately working toward a better quality of life for those affected by this condition.

## 1. Introduction

Sickle cell anemia (SCA) poses a myriad of challenges for those affected, and among the complications, gastrointestinal issues, particularly diarrhea, emerge as significant concerns. SCA is a hereditary blood disorder characterized by the presence of abnormal hemoglobin, leading to the formation of sickle-shaped red blood cells. Individuals with SCA often face complications such as vaso-occlusive crises and organ damage due to the impaired flow of blood. Gastrointestinal manifestations, including diarrhea, can exacerbate the overall health burden on individuals with SCA. Understanding and managing these challenges are essential components of comprehensive healthcare for individuals grappling with SCA, highlighting the need for specialized attention to gastrointestinal symptoms.^[1–3]^ Effectively addressing diarrhea in individuals with SCA requires a multifaceted approach that considers the unique aspects of the disorder. Gastrointestinal symptoms in SCA can result from various factors, such as infection, medications, or complications from vaso-occlusive crises. A tailored management strategy involves not only treating acute symptoms but also implementing preventive measures and promoting overall digestive health. This may include hydration strategies, dietary modifications, and close monitoring to detect and manage potential complications promptly. Moreover, healthcare providers must collaborate with individuals with SCA to develop personalized care plans that address the specific challenges posed by gastrointestinal symptoms, aiming for improved overall quality of life and better disease management.^[1]^

Sickle cell disease (SCD) is a condition affecting the entire body; the underlying reason for the manifestations would be blockage of the vessels by the sickled cells with added inflammation or infections. The attending physician who treats this underlying disease, along with preventive measures like avoiding dehydration and electrolyte imbalance, would automatically address the underlying mechanism. The general management of SCD holds good even for the gastrointestinal findings. The order in which organ systems are involved in the order of priority has to be considered. Managing gastrointestinal challenges, such as diarrhea, in individuals with SCA presents a unique set of considerations and concerns.^[1]^ SCA is a genetic blood disorder that affects the shape and function of red blood cells, leading to various complications.^[2]^ While the primary focus of SCA management is often on preventing and managing vaso-occlusive crises and pain, gastrointestinal issues like diarrhea can also significantly impact the quality of life for those living with this condition.^[3]^ SCA is a hereditary disorder characterized by the presence of abnormal hemoglobin, which causes red blood cells to become rigid and take on a crescent or sickle shape.^[4]^ This abnormal shape can obstruct blood vessels, leading to a wide range of complications, including pain crises, anemia, and organ damage. Diarrhea is a common gastrointestinal symptom in individuals with SCA.^[4]^ The exact cause of diarrhea in sickle cell patients can vary, but it may be related to factors like medications, infections, or the disease itself.^[5,6]^

Diarrhea can lead to dehydration, electrolyte imbalances, and further complications in people with SCA.^[7]^ Dehydration can exacerbate the risk of vaso-occlusive crises and other complications associated with the disease.^[8]^ Managing diarrhea in individuals with SCA requires a multifaceted approach.^[9]^ Proper hydration is crucial. Patients should drink plenty of fluids to prevent dehydration. In some cases, intravenous fluids may be necessary, especially if dehydration is severe. Maintaining a well-balanced diet is essential. A registered dietitian can help design a diet plan that meets the patient nutritional needs while considering any dietary restrictions or sensitivities. In some cases, antidiarrheal medications or medications to address the underlying cause of diarrhea may be prescribed under a healthcare provider guidance. Managing pain associated with diarrhea or sickle cell crises is important.^[10,11]^ Appropriate pain medications and pain management strategies should be part of the treatment plan.

Preventing diarrhea in sickle cell patients involves maintaining overall health and minimizing triggers.^[12]^ This may include avoiding certain foods or medications that can exacerbate gastrointestinal symptoms and managing stress, which can be a trigger for sickle cell crises. Individuals with SCA should have regular checkups with a healthcare provider who is experienced in managing the condition.^[13]^ This helps ensure early detection and management of any complications, including gastrointestinal issues. Educating individuals with SCA and their caregivers about the importance of proper hydration, nutrition, and recognizing the signs of dehydration and complications is essential for effective self-management.^[14]^ Managing diarrhea in individuals with SCA requires a comprehensive and multidisciplinary approach.^[15]^ Proper hydration, nutrition, and pain management are crucial components of care, and individuals living with this condition should work closely with healthcare providers to address their unique needs and challenges.

## 2. Pathophysiology of gastrointestinal challenges in SCA

The pathophysiology of gastrointestinal challenges, including diarrhea, in SCA is complex and multifactorial.^[16]^ It primarily stems from the fundamental genetic mutation that characterizes SCD, leading to the formation of abnormal hemoglobin, known as hemoglobin S (HbS). In SCA, individuals inherit 2 abnormal HbS genes. Under conditions of low oxygen or dehydration, HbS molecules tend to polymerize, forming long, rigid structures within the red blood cells.^[17–20]^ This results in the characteristic “sickling” of red blood cells, which leads to their altered shape and decreased flexibility. Sickled red blood cells have a tendency to adhere to the endothelial lining of blood vessels, leading to vaso-occlusion. These clumps of sickled cells can block small blood vessels, impairing blood flow to various tissues and organs, including the gastrointestinal tract. The reduced blood flow can cause ischemia (lack of oxygen) and damage to the intestines.^[21–25]^

The vaso-occlusive events in the gastrointestinal tract trigger an inflammatory response.^[26]^ Ischemia, a reduced blood supply, can lead to tissue damage and increased permeability of the intestinal wall. This can result in leakage of blood and protein into the intestines and the secretion of pro-inflammatory mediators. Endothelial cells lining the blood vessels can also be damaged by the adherence of sickled red blood cells. This endothelial injury can further contribute to inflammation, vasoconstriction, and compromised blood flow. SCA may also disrupt the balance of the gut microbiome, which can influence the gut function.^[27]^ Changes in the gut microbiota may contribute to gastrointestinal symptoms, including diarrhea. In cases where vaso-occlusion episodes resolve, the subsequent reperfusion of previously ischemic tissues can lead to additional tissue damage due to the release of reactive oxygen species and inflammatory mediators.^[28]^ SCA can lead to autonomic nervous system dysregulation, which may affect the motility and function of the gastrointestinal tract.^[29]^ This can result in delayed transit times and altered bowel habits, including diarrhea. Individuals with SCA may be more susceptible to infections and enteric pathogens due to immune system compromise.^[30]^ Infections and inflammatory responses to these pathogens can trigger or exacerbate gastrointestinal symptoms, including diarrhea.^[31]^ It is important to note that the severity and frequency of gastrointestinal complications, including diarrhea, can vary among individuals with SCA.^[32]^ Table 1 shows causes of diarrhea in SCA, Table 2 shows underlying mechanisms of diarrhea in SCA, Table 3 shows diagnosis of diarrhea in SCA and Table 4 shows management of diarrhea in SCA (author provided).

**Table 1:** Causes of diarrhea in SCA.

Causes	Description
Infection	Bacterial, viral, or parasitic infections affecting the gastrointestinal tract.
Medication side effects	Some medications used in SCA treatment may lead to gastrointestinal disturbances.
Ischemic colitis	Reduced blood flow to the colon, a common complication in SCA, can cause diarrhea.
Malabsorption	Impaired nutrient absorption due to SCA-related complications.

SCA = sickle cell anemia.

**Table 2:** Underlying mechanisms of diarrhea in SCA.

Mechanisms	Description
Vaso-occlusive crises	Episodes of blocked blood vessels affecting gut circulation.
Inflammatory response	Increased inflammation in the gastrointestinal tract due to SCA.
Altered gut microbiota	SCA-related factors may influence the balance of gut bacteria.
Medication interactions	Some drugs used in SCA treatment may disrupt normal gut function.

SCA = sickle cell anemia.

**Table 3:** Diagnosis of diarrhea in SCA.

Diagnostic methods	Description
Stool cultures	To identify infectious causes of diarrhea.
Colonoscopy	Allows direct visualization of the colon to assess for inflammation.
Blood tests	Evaluate for signs of inflammation, anemia, or nutritional deficiencies.
Imaging studies	Radiological assessments to identify structural or vascular abnormalities

SCA = sickle cell anemia.

**Table 4:** Management of diarrhea in SCA.

Management strategies	Description
Hydration	Adequate fluid intake to prevent dehydration, especially during crises.
Antibiotics	Targeted use if infection is identified as a cause of diarrhea.
Pain management	Addressing vaso-occlusive crises to reduce complications like ischemic colitis.
Nutritional support	Dietary modifications and supplements to manage malabsorption issues

SCA = sickle cell anemia.

## 3. Clinical presentation of diarrhea in SCA

The clinical presentation of gastrointestinal challenges, particularly diarrhea, in individuals with SCA can vary in severity and frequency.^[33]^ These challenges often result from the complex pathophysiological processes that affect the gastrointestinal tract. Diarrhea in SCA can be intermittent or chronic. Some individuals may experience occasional episodes, while others may have persistent diarrhea.^[34]^ The severity can range from mild to severe. Diarrhea in SCA typically presents with loose or watery stools. The consistency of stools may vary, and they may be accompanied by an increased urgency to have a bowel movement. Many individuals with SCA experience abdominal pain in conjunction with diarrhea.^[35]^ The pain can range from mild discomfort to severe cramping and may be localized or diffuse. Some patients may complain of abdominal bloating and increased gas production along with diarrhea. In severe cases, diarrhea in SCA can lead to gastrointestinal bleeding, which may result in the passage of blood in the stools (hematochezia).^[36]^ Chronic and severe diarrhea can lead to malabsorption of nutrients, resulting in weight loss and malnutrition. Frequent or severe diarrhea episodes can lead to dehydration, which may manifest with symptoms such as dry mouth, increased thirst, reduced urine output, and lethargy. Diarrhea can have a significant impact on the overall quality of life for individuals with SCA, affecting daily activities and social functioning.^[37]^ It important to note that while diarrhea is a common gastrointestinal symptom in SCA, it can also be caused by other factors, such as infections, medications, or dietary choices. Therefore, a thorough evaluation by a healthcare professional is necessary to determine the specific cause and appropriate management. Diarrhea in SCA should not be ignored, as it can lead to complications such as malnutrition, electrolyte imbalances, and further exacerbation of the underlying disease.^[38]^ Effective management strategies, as well as patient education, are crucial to address this aspect of the disease and improve the well-being of affected individuals.

## 4. Diagnosis and evaluation of diarrhea in SCA

The diagnosis and evaluation of diarrhea in individuals with SCA involve a comprehensive approach to identify the underlying causes and determine appropriate management strategies.^[39]^ Begin with a thorough medical history and physical examination to assess the patient overall health, SCA status, and any other relevant medical conditions. Document the frequency, duration, and severity of diarrhea episodes.^[40]^ Inquire about associated symptoms such as abdominal pain, bloating, and blood in stools. Perform routine blood tests, including a complete blood count, to assess hemoglobin levels and any potential anemia. Check for evidence of inflammation with tests like C-reactive protein or erythrocyte sedimentation rate. Evaluate electrolyte imbalances, particularly potassium and magnesium levels, which can be affected by chronic diarrhea. Conduct stool tests to assess for infection or inflammation. This may include tests for bacterial, viral, or parasitic pathogens, as well as fecal calprotectin, a marker of intestinal inflammation. Consider endoscopic procedures such as colonoscopy or upper endoscopy to visualize and assess the gastrointestinal tract directly. These procedures can help identify structural abnormalities, bleeding sources, or inflammatory conditions. If indicated, use imaging studies like abdominal ultrasound, computed tomography (CT) scans, or magnetic resonance imaging to identify anatomical issues in the gastrointestinal tract. In cases where endoscopic procedures are performed, biopsies of the intestinal tissue can be taken to assess for histopathological changes, including inflammation, ulcers, or other abnormalities. Assess gastrointestinal motility through specialized studies if delayed transit times or motility issues are suspected. Consider genetic testing to confirm the diagnosis of SCA and assess the specific mutations present in the HBB gene.

Depending on the findings, consult with specialists such as gastroenterologists, hematologists, or nutritionists to provide expertise in managing gastrointestinal and hematological aspects.^[41]^ Educate the patient about the nature of SCA and its gastrointestinal complications, emphasizing the importance of hydration and proper dietary choices.^[42]^ Develop a personalized treatment plan based on the identified causes of diarrhea. This may include dietary modifications, medication to manage symptoms, and addressing any underlying issues. Diarrhea in individuals with SCA can result from a combination of factors, including the disease itself, infections, dietary choices, and medications.^[43]^ The diagnostic and evaluation process aims to uncover the specific contributors to diarrhea, allowing for a more targeted and effective management strategy. Close collaboration among healthcare professionals is often required to address the complex nature of this condition.

## 5. Differential diagnosis of diarrhea in SCA

When evaluating diarrhea in individuals with SCA, it important to consider a broad range of potential causes, as not all cases of diarrhea are directly related to the underlying condition. Vaso-occlusive crises can lead to ischemia in the gut, causing abdominal pain and diarrhea.^[44]^ Differentiating this from other causes of diarrhea is important. Gastrointestinal infections, such as viral, bacterial, or parasitic infections, can cause acute diarrhea. SCA patients may be more susceptible to infections due to compromised immunity.

Some medications used in the treatment of SCA, such as hydroxyurea or pain management drugs, can have gastrointestinal side effects, including diarrhea.^[45]^ Dietary choices, food intolerances, or allergies can lead to gastrointestinal symptoms, including diarrhea. Malabsorption of specific nutrients may occur, contributing to diarrhea. Conditions like Crohn disease and ulcerative colitis can present with chronic diarrhea and abdominal pain. They may coexist with SCA. IBS is characterized by abdominal pain, changes in bowel habits, and diarrhea. It can be triggered or exacerbated by stress, which is a common experience in individuals with SCA. Celiac disease, an autoimmune condition triggered by gluten consumption, can lead to chronic diarrhea and malabsorption of nutrients. Various medications unrelated to SCA can lead to diarrhea as a side effect, such as antibiotics, laxatives, and some antihypertensive drugs.^[46]^ Lactose intolerance can cause diarrhea in individuals who cannot digest lactose, a sugar found in milk and dairy products. Conditions like peptic ulcers, esophagitis, or vascular malformations can lead to gastrointestinal bleeding, which may result in bloody stools and diarrhea. Conditions like pancreatic insufficiency, celiac disease, or short bowel syndrome can result in malabsorption of nutrients and diarrhea. Ingesting toxic substances, such as certain chemicals or spoiled food, can lead to acute diarrhea. Autoimmune disorders, such as systemic lupus erythematosus (SLE), can affect the gastrointestinal tract and cause diarrhea.^[47]^ Conditions like hyperthyroidism or diabetes can lead to changes in bowel habits, including diarrhea. Psychological factors, including stress and anxiety, can exacerbate gastrointestinal symptoms, including diarrhea.^[48]^

To determine the specific cause of diarrhea in individuals with SCA, a comprehensive evaluation is necessary.^[49]^ This may include a thorough medical history, physical examination, laboratory tests, imaging, endoscopic procedures, and consultation with specialists. Differential diagnosis is crucial to provide appropriate and effective management tailored to the underlying cause of diarrhea.

## 6. Management strategies of diarrhea in SCA

Managing diarrhea in individuals with SCA involves a multifaceted approach that addresses both the underlying disease and the specific causes of diarrhea.^[50]^ Adequate hydration is crucial to prevent dehydration, which can exacerbate the symptoms of diarrhea. Encourage increased fluid intake. Monitor and correct electrolyte imbalances, particularly potassium and magnesium, which can be lost due to diarrhea. Work with a registered dietitian to develop a dietary plan that is tailored to the individual needs. In some cases, a low-fiber or lactose-free diet may be recommended. Avoid triggering foods or beverages, such as caffeine, spicy foods, and high-fiber items that may exacerbate diarrhea. Antidiarrheal medications, such as loperamide (Imodium), may be prescribed to help control diarrhea.^[51]^ However, their use should be carefully monitored by a healthcare professional. Probiotics or prebiotics may be considered to help restore and maintain a healthy gut microbiome.

## 7. Management of underlying SCA

Ensure that the underlying SCA is well-managed.^[52]^ This may involve regular blood transfusions, hydroxyurea therapy, or other disease-modifying treatments as prescribed by a hematologist. Promptly identify and treat any infections that may be causing or exacerbating diarrhea. This includes using appropriate antibiotics or antiviral medications. Manage sickle cell-related pain effectively, as uncontrolled pain can exacerbate gastrointestinal symptoms.^[42]^ Pain medications should be used as prescribed. Provide resources and support for individuals to manage stress and anxiety, as these emotional factors can influence gastrointestinal symptoms. Educate the patient about their condition and the importance of managing diarrhea. Encourage lifestyle modifications, including stress reduction, regular exercise, and adequate sleep. Schedule regular follow-up appointments with healthcare providers to monitor the effectiveness of the management plan, assess for complications, and make any necessary adjustments. Offer psychological support and counseling for individuals dealing with the physical and emotional challenges of SCA and its associated symptoms, including diarrhea.^[53]^ In cases of severe complications, such as intestinal infarction or intractable bleeding, surgical intervention may be necessary. This may include partial bowel resection or other procedures.

It is important to note that the management of diarrhea in SCA should be individualized based on the specific needs and causes of diarrhea for each patient.^[10]^ Healthcare professionals, including hematologists and gastroenterologists, should collaborate to develop and implement a comprehensive and tailored treatment plan.^[54]^ Regular communication between the patient and their healthcare team is essential to monitor progress and adjust the management strategies as needed.^[55]^

## 8. Complications and risk factors of diarrhea in SCA

Diarrhea in individuals with SCA can lead to various complications and is influenced by specific risk factors.^[16]^ Understanding these complications and risk factors is crucial for the effective management of this condition. Chronic or severe diarrhea can result in dehydration due to excessive fluid loss. Dehydration can lead to symptoms such as dry mouth, increased thirst, reduced urine output, and, in severe cases, electrolyte imbalances.^[56]^ Prolonged diarrhea can cause malabsorption of essential nutrients, including proteins, vitamins, and minerals. This can result in malnutrition, weight loss, and weakness. Diarrhea can lead to the loss of electrolytes, particularly potassium and magnesium. Electrolyte imbalances can have various health implications, including cardiac arrhythmias. Malabsorption associated with chronic diarrhea may lead to specific nutrient deficiencies, such as iron-deficiency anemia or vitamin deficiencies.^[57]^ Diarrhea, especially if it causes dehydration or electrolyte imbalances, can exacerbate the underlying SCA, potentially leading to vaso-occlusive crises, pain, and other complications related to the disease.^[58]^ Chronic diarrhea may increase the risk of gastrointestinal infections, which can further complicate the clinical picture and worsen the symptoms.

## 9. Risk factors

The specific genotype of SCD (HbSS, HbSC, HbSβ0-thalassemia, etc) can influence the severity and frequency of gastrointestinal complications, including diarrhea. Psychological factors, such as stress and anxiety, can trigger or exacerbate gastrointestinal symptoms, including diarrhea. Diet plays a significant role in the development of diarrhea. Foods that are poorly tolerated, high in fiber, or have known gastrointestinal irritants may contribute to diarrhea. Some medications used in the management of SCA, such as hydroxyurea or pain medications, can have gastrointestinal side effects, including diarrhea. Infections, particularly viral or bacterial infections, can trigger or worsen diarrhea in individuals with SCA.^[45]^ Their compromised immune system can make them more susceptible to infections. Ongoing inflammation within the gastrointestinal tract, often due to repeated vaso-occlusive crises and ischemia, can contribute to the development of diarrhea.^[59]^ Poor nutritional status, including malnutrition, can increase the risk of developing diarrhea and worsen its consequences. It essential to conduct a comprehensive evaluation to determine the specific causes of diarrhea in individuals with SCA and identify any underlying risk factors.^[60]^ Tailored management strategies should address both the underlying disease and the specific contributing factors to minimize complications and improve the individual quality of life. Regular follow-up and communication with healthcare providers are critical to monitor and adjust the management plan as needed.

## 10. Patient education for diarrhea in SCA

Patient education is a vital component of managing diarrhea in individuals with SCA.^[61]^ Proper education can empower patients to understand their condition, make informed choices, and actively participate in their healthcare. Explain the basics of SCA, including its genetic nature and the impact on red blood cells and blood circulation.^[62]^ Discuss the gastrointestinal complications associated with SCA, emphasizing that diarrhea is a common symptom. Help patients recognize the signs of diarrhea, including changes in stool frequency, consistency, and associated symptoms like abdominal pain or bloating. Describe the potential causes of diarrhea in SCA, including the disease itself, infections, medications, stress, dietary choices, and other risk factors.^[36]^ Stress the importance of maintaining good hydration to prevent dehydration, especially when experiencing diarrhea. Encourage regular fluid intake and consider the use of oral rehydration solutions as needed. Provide dietary recommendations, such as a balanced and fiber-appropriate diet, and suggest avoiding foods or beverages that may exacerbate diarrhea. Explain the use of antidiarrheal medications if prescribed and the importance of using them as directed. Emphasize the necessity of regular follow-up appointments with healthcare providers to monitor symptoms, adjust the management plan, and address any complications. Discuss stress reduction techniques, as stress can trigger or worsen gastrointestinal symptoms. Educate patients on infection prevention strategies, such as practicing good hand hygiene and avoiding contact with sick individuals.^[63]^ Stress the importance of adhering to the prescribed medications for SCA management and pain control.^[64]^ If necessary, involve a registered dietitian to provide guidance on maintaining proper nutrition and addressing dietary intolerances. Educate patients on recognizing signs of severe complications and when to seek immediate medical attention, such as for signs of intestinal infarction. Highlight the availability of psychological support services to help patients cope with the emotional aspects of living with SCA and its associated symptoms.^[65]^ Reinforce the goal of improving the patient overall quality of life and well-being by effectively managing their symptoms. Stress the importance of individualized care, as the causes and severity of diarrhea can vary among individuals with SCA. Patient education should be an ongoing process, and healthcare providers should encourage open communication with patients to address their questions and concerns. Additionally, patient education materials, support groups, and counseling services can be valuable resources to enhance the patient understanding of their condition and its management.

## 11. Probiotics in the treatment of diarrhea in SCA

Diarrhea in individuals with SCA can result from various factors, including infections, altered gut microbiota, and medication side effects. Probiotics, live microorganisms with potential health benefits, have gained attention for their role in promoting gastrointestinal health. In the context of SCA-related diarrhea, exploring the use of probiotics as a complementary approach is of interest. Probiotics may offer several potential benefits in managing diarrhea in individuals with SCA. SCA-related complications and frequent healthcare interventions can disrupt the balance of gut bacteria. Probiotics may aid in restoring healthy gut microbiota, potentially reducing the frequency and severity of diarrhea episodes. Probiotics have immunomodulatory properties, which may help regulate the immune response in the gastrointestinal tract. This can be beneficial in addressing inflammatory processes contributing to diarrhea in SCA. By promoting a balanced and diverse gut microbiota, probiotics may help prevent or reduce the impact of infectious causes of diarrhea, a common concern in individuals with compromised immune systems such as those with SCA. Before incorporating probiotics into the treatment plan, individuals with SCA should consult their healthcare providers. Probiotics may interact with medications or other treatments, and personalized advice is essential. Different probiotic strains may have varying effects. Research on specific strains’ efficacy in addressing SCA-related diarrhea is limited, emphasizing the need for more targeted studies to establish optimal recommendations. Continuous monitoring of symptoms and an individualized approach to probiotic use are crucial. Adjustments to the probiotic regimen may be necessary based on the individual response and evolving health status.^[63,64]^

## 12. Future directions and research for diarrhea in SCA

Research into diarrhea and its management in individuals with SCA is an evolving field, and ongoing studies aim to improve our understanding of the condition and develop more effective treatment strategies.^[46,66]^ Further research is needed to better understand the underlying mechanisms responsible for diarrhea in individuals with SCA. This includes investigating the role of sickling, inflammation, and other factors contributing to gastrointestinal complications. Exploring genetic and molecular markers associated with the severity and frequency of gastrointestinal symptoms in different genotypes of SCA can help personalize treatment approaches.^[67]^ The development of specific biomarkers for diagnosing and monitoring gastrointestinal complications can improve early intervention and treatment. Research should focus on the development of targeted therapies that address the specific causes of diarrhea in SCA, such as drugs that can reduce inflammation or enhance intestinal motility. Investigating the role of probiotics and their impact on the gut microbiome in individuals with SCA can provide insights into managing gastrointestinal symptoms. Conducting well-designed clinical trials to evaluate the efficacy and safety of new medications or interventions for managing diarrhea in SCA. Collecting patient-reported outcomes and quality-of-life data to understand the impact of diarrhea on individuals with SCA and to guide the development of patient-centered interventions.^[68]^ Studying strategies to prevent gastrointestinal complications, such as the use of hydroxyurea, regular blood transfusions, or other disease-modifying therapies, can help reduce the frequency and severity of diarrhea. Investigating the psychological impact of chronic diarrhea in individuals with SCA and developing interventions to support their mental well-being. Further research into dietary modifications and nutritional support, including personalized dietary plans, to alleviate diarrhea and improve overall health. Evaluating the effectiveness of telemedicine and remote monitoring for managing gastrointestinal complications in individuals with SCA, especially in remote or underserved areas.^[69]^ Exploring the burden of diarrhea in SCA on a global scale and identifying strategies to improve care, especially in regions with limited resources. Encouraging collaboration between hematologists, gastroenterologists, nutritionists, and psychologists to provide comprehensive care for individuals with SCA.^[70]^

Research in these areas can lead to a better understanding of the causes, management, and long-term outcomes of diarrhea in SCA, ultimately improving the quality of life for those affected by this condition. Patients, healthcare professionals, and researchers all play important roles in advancing our knowledge and developing more effective strategies for managing gastrointestinal challenges in SCA.

## 13. Conclusion

In conclusion, managing gastrointestinal challenges, specifically diarrhea, in individuals with SCA is a complex and multifaceted endeavor. This publication has explored various aspects of this condition, from its pathophysiology and clinical presentation to its diagnosis, complications, risk factors, and management strategies. It has also highlighted the importance of patient education and the need for ongoing research to advance our understanding of this critical issue.

Gastrointestinal complications, including diarrhea, can significantly impact the quality of life of individuals with SCA. Effective management involves a combination of strategies, such as hydration, dietary modifications, medications, and addressing the underlying disease. Patient education is a key component of this management, enabling individuals to actively participate in their care, recognize symptoms, and make informed choices to enhance their well-being.

## Author contributions

**Conceptualization:** Emmanuel Ifeanyi Obeagu.

**Methodology:** Emmanuel Ifeanyi Obeagu, Getrude Uzoma Obeagu.

**Supervision:** Emmanuel Ifeanyi Obeagu.

**Validation:** Emmanuel Ifeanyi Obeagu.

**Visualization:** Emmanuel Ifeanyi Obeagu.

**Writing – original draft:** Emmanuel Ifeanyi Obeagu, Getrude Uzoma Obeagu.

**Writing – review & editing:** Emmanuel Ifeanyi Obeagu, Getrude Uzoma Obeagu.
